# Prognostic value of epithelial-mesenchymal transition markers in clear cell renal cell carcinoma

**DOI:** 10.18632/aging.102660

**Published:** 2020-01-08

**Authors:** Hua Xu, Wen-Hao Xu, Fei Ren, Jun Wang, Hong-Kai Wang, Da-Long Cao, Guo-Hai Shi, Yuan-Yuan Qu, Hai-Liang Zhang, Ding-Wei Ye

**Affiliations:** 1 Department of Urology, Fudan University Shanghai Cancer Center, Shanghai 200032, China; 2 Department of Oncology, Shanghai Medical College, Fudan University, Shanghai 200032, China; 3 Department of Pathology, Fudan University Shanghai Cancer Center, Shanghai 200032, China

**Keywords:** clear cell renal cell carcinoma, epithelial-to-mesenchymal transition, prognosis, tumor microenvironment, predictive model

## Abstract

Epithelial-to-mesenchymal transition (EMT) is important in tumor invasiveness and metastasis. We aimed to determine prognostic value of six key EMT markers (*CDH1*, *CDH2*, *SNAI1*, *SNAI2*, *VIM*, *TWIST1*) in clear cell renal cell carcinoma (ccRCC). A total of 533 ccRCC patients with RNASeq data from The Cancer Genome Atlas (TCGA) cohort were included for analysis. Gene expression of these EMT markers was compared between tumor and normal tissues based on Oncomine database and TCGA cohort. Their correlations with progression-free survival (PFS) and overall survival (OS) were also examined in both TCGA cohort and FUSCC (Fudan University Shanghai Cancer Center) cohort. Cox proportional hazards regression model and Kaplan-Meier plot were used to assess the relative factors. Functional enrichment analyses were utilized to describe biologic function annotations and significantly involved hallmarks pathways of each gene. We found that Epithelial marker, *CDH1* expression was lower, while mesenchymal markers (*CDH2*, *SNAI1*, *VIM*, *TWIST1*) expression was higher in ccRCC primary tumors. In the TCGA cohort, we found that patients with higher expression of *VIM*, *TWIST1* or lower expression of *CDH1* had worse prognosis. Further, in the FUSCC cohort, we confirmed the predictive ability of mesenchymal markers and epithelial marker expression in PFS and OS of ccRCC patients. After generating Cox regression models, EMT markers (*CDH1*, *SNAI1*, *VIM*, and *TWIST1*) were independent prognostic factors of both PFS and OS in ccRCC patients. Our preliminary EMT prediction model can facilitate further screening of EMT biomarkers and cast a better understanding of EMT gene function in ccRCC.

## INTRODUCTION

Kidney cancer is one of the most common urological tumors worldwide, and nearly 73,820 new cases and 14,770 deaths were estimated in the United States in 2019 [1]. The incidence and mortality of kidney cancer is also increasing in China with estimated 66,800 new cases and 23,400 deaths in 2015 [2]. Clear cell renal cell carcinoma (ccRCC), a major subtype of kidney cancer is the most common type of renal cell carcinoma (RCC) in adults. It is one of the most lethal urological tumors with worldwide mortality of about 90,000 annually based on the WHO statistics [3]. Thanks to early detection and technology improvement, an increase in the proportion of patients with early stage RCC (T1) from 40% before 1993 to 60% in 2004 is observed according to data from the National Cancer Database (NCDB) [4]. Besides, minimally invasive technologies as well as active surveillance have also been improved. However, the five-year overall survival for RCC has just increased from 57% in the late 1980s to 70% recently [5], and RCC patient prognosis may be unchanged [6]. Better prediction of RCC patients after surgery would help make a more suitable and beneficial treatment plan for them.

Tumor stage is the most powerful, but still a relatively crude predictor of patients survival with ccRCC [7]. Since 2001, several mathematical models and nomograms have also been developed for both localized and metastatic ccRCC using factors such as stage, symptoms, performance status (PS), and tumor size [8–10]. Application of these algorithms contributes to fewer radiographic imaging and blood tests for patients who are predicted to have a low risk of recurrence after surgery, and suggestion of adjuvant therapy for high-risk patients. The limitations of these prognostic algorithms and the varied response to surgery suggest molecular diversity in ccRCC. Molecular features involved prognostic algorithms might improve ccRCC prognosis prediction.

Epithelial-to-mesenchymal transition (EMT), first described in the 1980s, is involved in physiological embryogenesis as well as in some pathological processes, such as solid organ fibrosis [11]. EMT is defined as a process that epithelial cells develop a mesenchymal phenotype and acquire motility with loss of their polarity and barrier integrity [12]. EMT also plays a crucial role in tumor invasiveness and metastasis [13], including ccRCC. Previously, a few studies focused on the expression of EMT related genes in RCC mostly at the protein level using immunohistochemistry [14–16]. In the study, we focus on mRNA level to assess prognostic implications of EMT in ccRCC. We include six key EMT related genes and construct a five-gene signature using data from The Cancer Genome Atlas (TCGA) databases. We validated this signature in a cohort of ccRCC patients who underwent nephrectomy at Fudan University Shanghai Cancer Center (FUSCC).

## RESULTS

This study consisted of three stages. In the first stage, we assessed differential expressed *CDH1, CDH2, SNAI1, SNAI2, VIM, TWIST1* in transcriptional and protein level according to datasets hosted on the Oncomine and TCGA platform; in the second stage, survival analysis based on distinct comparison expression of six hub genes have been evaluated in TCGA cohort and validated in FUSCC cohort; in the third stage, significantly involved hub genes were selected, and functional annotation of hub genes were elaborated.

### Differential expression of EMT related genes in ccRCC patients

**Figure 1:**
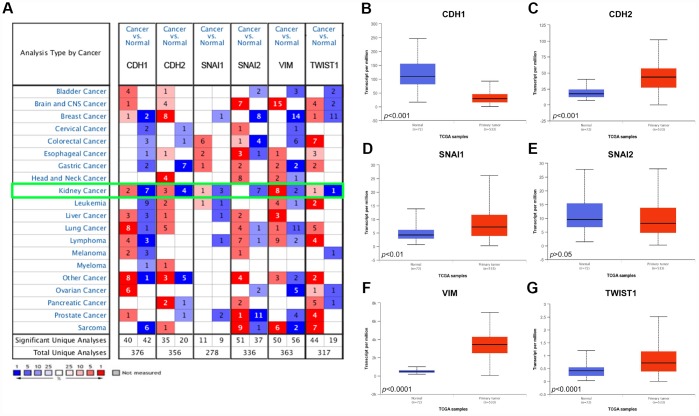
**Analysis of the six EMT related genes in Oncomine database and TCGA database.** (**A**) The Oncomine database was queried for the expression of *CDH1*, *CDH2*, *SNAI1*, *SNAI2*, *VIM*, and *TWIST1* in the available datasets based on the following criteria: 1) “Cancer Type”; 2) “Gene: *CDH1*, *CDH2*, *SNAI1*, *SNAI2*, *VIM*, or *TWIST1*”; 3) “Data Type: mRNA”; 4) “Analysis Type: Cancer vs Normal Analysis”, and 5) Threshold Setting Condition (*p*<0.001, fold change >2, gene rank = top 10%). The 'red cells' represents gene overexpression and the 'blue cells' represent gene underexpression. The color intensity equals the percentile, i.e. Top 1%, 5%, or 10% significantly over- or underexpressed (see the legend below the grid). We found that *CDH1* and *SNAI2* was underexpressed in the kidney cancer vs normal datasets, while VIM was highly overexpressed. (**B**–**G**) Differential mRNA expression of six EMT related genes in clear cell renal cell carcinoma (ccRCC) tumor samples and adjacent normal tissues from TCGA. Epithelial marker *CDH1* mRNA expression was significantly lower in tumor samples compared with adjacent normal tissues (**B**); Most mesenchymal markers (*CDH2*, *SNAI1*, *VIM*, and *TWIST1*) mRNA expression was elevated in tumor samples compared with adjacent normal tissues (**C**–**G**).

As shown in Figure 1A, Oncomine datasets demonstrated *CDH1, CDH2, SNAI1, SNAI2, VIM, TWIST1* in 20 types of cancers between tumor and normal tissues. Transcriptional expressions of VIM were significantly elevated in cancer tissue compared with normal tissues, while expression of CDH1, a key epithelial marker, was significantly decreased in cancer tissue in multiple datasets.

We further compared the mRNA expression of *CDH1, CDH2, SNAI1, SNAI2, VIM, TWIST1* between ccRCC tumor samples and adjacent normal tissues respectively based on RNA-sequence data from TCGA database. Consistent with Oncomine data, CDH1 expression was lower in ccRCC primary tumors in comparison with adjacent normal tissues (Figure 1B). However, expression of mesenchymal markers, *CDH2, SNAI1, VIM, TWIST1*, were significantly higher in ccRCC primary tumors (Figure 1C–1G).

### Neighbor genes and hierarchical partitioning of hub genes from TCGA

As was shown in Supplementary Figure 1, network of *CDH1, CDH2, SNAI1, SNAI2, VIM, TWIST1* and their 49 frequently genetic altered neighbor genes was integrated and constructed using cBioPortal.

### Demographic and clinical characteristics of ccRCC patients in TCGA and FUSCC cohorts

The TCGA cohort comprised 337 (65.31%) male patients and 179 (34.69%) female patients. The median age of the 516 ccRCC patients was 60.5 years, with a range from 26 to 90 years. Information of TNM stage, AJCC stage, ISUP grade, laterality was shown in Table 1. The median follow-up time was 40.6 months and 172 (33.33%) patients died during follow-up. Besides, 114 (20.09%) patients developed progression or recurrence after surgery.

**Table 1:** Patient characteristics in TCGA cohort and FUSCC cohort.

**Clinicopathologic characteristics**	**TCGA cohort, N=516**		**FUSCC cohort, N=367**	
	**Median or number**	**Range or percentage (%)**	**Median or number**	**Range or percentage (%)**
**Age (years)**	60.5	26-90	56	21-86
**Follow-up length (months)**	40.6	0.1-151.2	60	7-110
**Gender**				
Male	337	65.31	248	67.57
Female	179	34.69	119	32.43
**Living status**				
Dead	172	33.33	135	36.78
Alive	344	66.67	232	63.22
**Progression**				
Yes	114	22.09	196	53.41
No	402	77.91	171	46.59
**ISUP grade**				
I-II	234	45.35	175	47.68
II-IV	277	53.68	192	52.32
Unclear	5	0.97		
**pT stage**				
T1	263	50.97	224	61.04
T2	67	12.98	66	17.98
T3	175	33.91	70	19.07
T4	11	2.13	7	1.91
**pN stage**				
N0	238	46.12	320	87.19
N1	15	2.91	47	12.81
Nx	263	50.97		
**M Stage**				
M0	413	80.04	321	89.1
M1	77	14.92	40	10.9
Mx	26	5.04	0	0
**AJCC stage**				
Stage I	257	49.81	218	59.4
Stage II	55	10.66	55	14.99
Stage III	122	23.64	40	10.9
Stage IV	82	15.89	54	14.71
**Laterality**				
Left	242	46.90	182	49.59
Right	273	52.91	185	50.41
Bilateral	1	0.19		
**BMI**				
<25			231	62.94
>25			136	37.06

Abbreviation: TCGA: The Cancer Genome Atlas; FUSCC: Fudan University Shanghai Cancer Center; ISUP: The International Society of Urological Pathology; AJCC: American Joint Committee on Cancer; BMI: body mass index.

The FUSCC cohort consisted of 248 (67.57%) male patients and 119 (32.43%) female patients. The median age of the 367 ccRCC patients was 56 years, with a range from 21 to 86 years. The detailed clinical data are shown in Table 1. During follow-up (median: 60 months), 135 (36.78%) patients died and 196 (53.41%) patients developed progression or recurrence after surgery.

### Prognostic value of expression of EMT related genes in ccRCC patients

**Figure 2:**
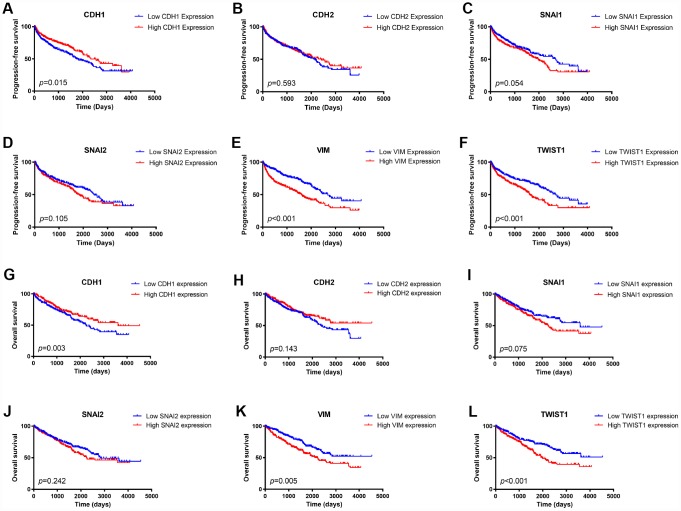
**Kaplan Meier survival plot of ccRCC patients in TCGA database according to high and low mRNA expression of six EMT related genes.** *CDH1* mRNA expression was associated with both worse progression-free survival (*p*=0.015) and worse overall survival (*p*=0.003) of ccRCC patients (**A**, **G**); *CDH2* mRNA expression was not an indicator of either progression-free survival (*p*=0.593) or overall survival (*p*=0.075) of ccRCC patients (**B**, **H**); Higher *SNAI1* mRNA expression was moderately associated with both worse progression-free survival (*p*=0.054) and worse overall survival (*p*=0.010) of ccRCC patients (**C**, **I**); *SNAI2* mRNA expression was not an indicator of either progression-free survival (*p*=0.105) or overall survival (*p*=0.242) of ccRCC patients (**D**, **J**); Higher *VIM* mRNA expression was associated with both worse progression-free survival (*p*<0.001) and worse overall survival (*p*=0.005) of ccRCC patients (**E**, **K**); Higher *TWIST1* mRNA expression was associated with both worse progression-free survival (*p*<0.001) and worse overall survival (*p*<0.001) of ccRCC patients (**F**, **L**).

We first examined the prognostic value of mRNA expression of these six EMT key markers in the TCGA cohort. A Kaplan-Meier plot indicated that patients with lower expression of epithelial marker, *CDH1*, had worse PFS (Figure 2A) as well as OS (Figure 2G). Besides, patients with higher expression of mesenchymal markers, *VIM, or TWIST1*, had both worse PFS (Figure 2E, 2F) and worse OS (Figure 2K–2J). Higher *SNAI1* expression was also associated with moderately worse PFS (Figure 2C) and OS (Figure 2I).

**Figure 3:**
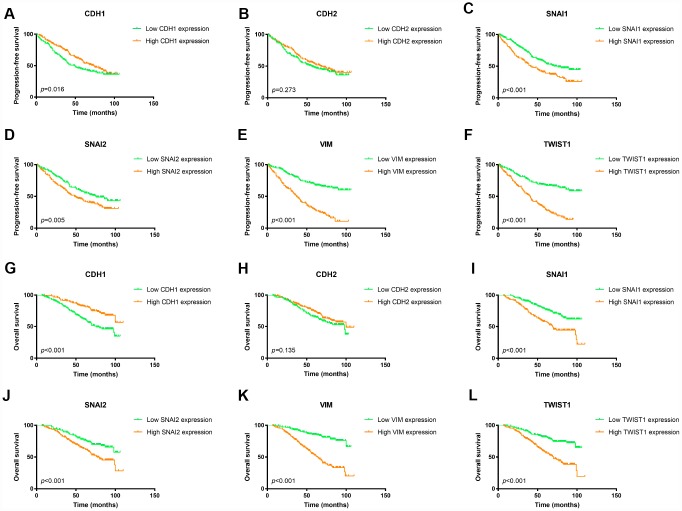
**Kaplan Meier survival plot of ccRCC patients in FUSCC cohort according to high and low mRNA expression of six EMT related genes.** Lower *CDH1* mRNA expression was associated with both worse progression-free survival (*p*=0.016) and worse overall survival (*p*<0.001) of ccRCC patients (**A**, **G**); *CDH2* mRNA expression was not an indicator of either progression-free survival (*p*=0.288) or overall survival (*p*=0.202) of ccRCC patients (**B**, **H**); Higher *SNAI1* mRNA expression was associated with both worse progression-free survival (*p*<0.001) and worse overall survival (*p*<0.001) of ccRCC patients (**C**, **I**); Higher *SNAI2* mRNA expression was associated with both worse progression-free survival (*p*=0.005) and worse overall survival (*p*<0.001) of ccRCC patients (**D**, **J**); Higher *VIM* mRNA expression was associated with both worse progression-free survival (*p*<0.001) and worse overall survival (*p*<0.001) of ccRCC patients (**E**, **K**); Higher *TWIST1* mRNA expression was associated with both worse progression-free survival (*p*<0.001) and worse overall survival (*p*<0.001) of ccRCC patients (**F**, **L**).

To confirm the prognostic value of these EMT related genes, we validated it in the FUSCC ccRCC cohort. As shown in Figure 3, patients with lower expression of CDH1 would have worse PFS as well as OS (Figure 3A and 3G). However, elevated expression of mesenchymal markers, *SNAI1, SNAI2, VIM, and TWIST1,* were associated with both worse PFS and worse OS (Figure 3C–3F, 3I–3L).

### Integrated prognostic and diagnostic model

**Figure 4:**
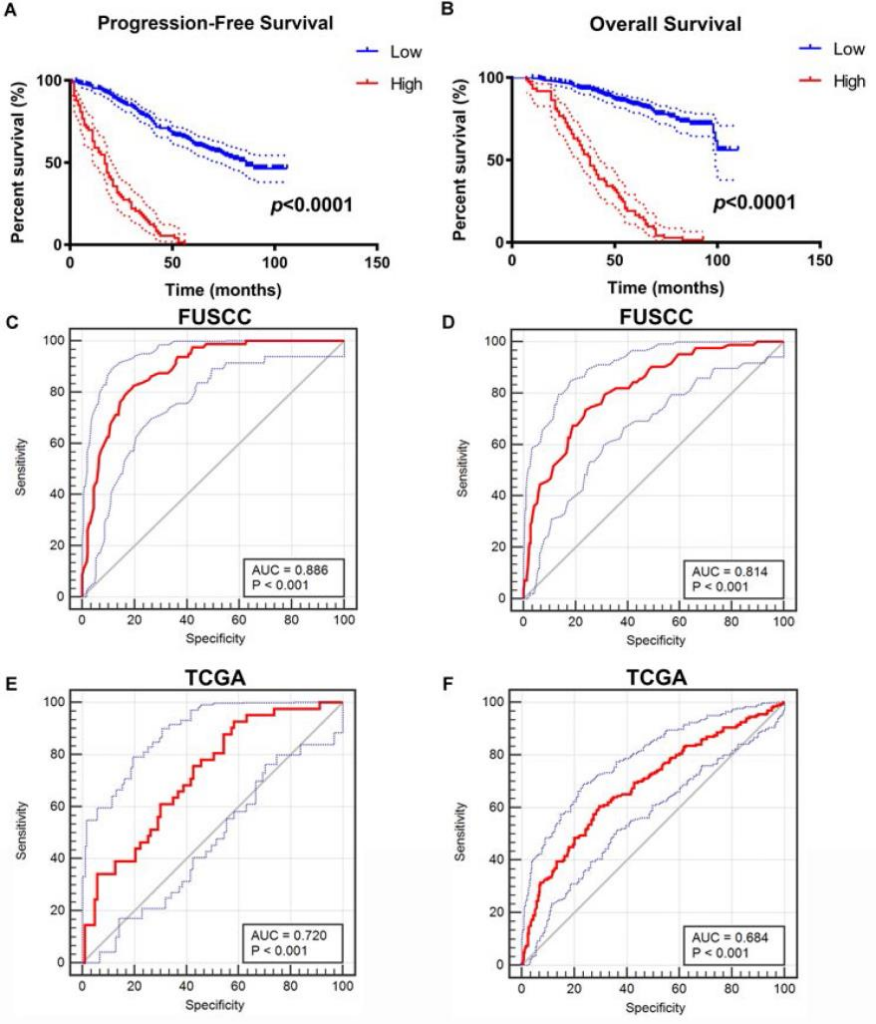
**Construction and internal validation of integrated prognostic and diagnostic model.** All significant clinicopathologic parameters and gene expression profiles was integrated in the Cox regression models, which indicated this formula: = -0.708×*CDH1* expression (ref. Low) + 1.360×*SNAI1* expression (ref. Low) + 1.905×*VIM* expression (ref. Low) + 2.179×*TWIST1* expression (ref. Low) + 1.274×T stage (ref. T1-T2) + 1.919×M stage (ref. M0) + 2.021×AJCC stage (ref. I-II) + 2.013×ISUP grade (ref. 1-2) for PFS (**A**), and another formula: = -0.564×*CDH1* expression (ref. Low) + 1.532×*SNAI1* expression (ref. Low) + 1.804×*VIM* expression (ref. Low) + 1.714×*TWIST1* expression (ref. Low) + 1.226×T stage (ref. T1-T2) + 1.778×M stage (ref. M0) + 2.515×AJCC stage (ref. I-II) + 1.954×ISUP grade (ref. 1-2) for OS (**B**). The Kaplan–Meier method was used to determine the significant survival outcomes (PFS: p<0.0001; OS: p<0.0001). ROC curves were generated to validate the ability of the logistic model to predict prognosis. The AUC index for the integrated model were 0.886 for PFS (p<0.001) (**C**) and 0.814 for OS (p<0.001) (**D**). (**E**, **F**) External validation of integrated model using TCGA cohorts. ROC curves were constructed to perform external validation using clinicopathological parameters and mRNA expression profiles from TCGA cohort. The AUC index for the integrated model were 0.720 for PFS (p<0.001) (**E**) and 0.684 for OS (p<0.001) (**F**).

After integrating all the significant clinicopathological parameters and gene expression profiles in the Cox regression models (Table 2), we generated the formula: = -0.708×*CDH1* expression (ref. Low) + 1.360×*SNAI1* expression (ref. Low) + 1.905×*VIM* expression (ref. Low) + 2.179×*TWIST1* expression (ref. Low) + 1.274×T stage (ref. T1-T2) + 1.919×M stage (ref. M0) + 2.021×AJCC stage (ref. I-II) + 2.013×ISUP grade (ref. 1-2) for PFS, and another formula: = -0.564×*CDH1* expression (ref. Low) + 1.532×*SNAI1* expression (ref. Low) + 1.804×*VIM* expression (ref. Low) + 1.714×*TWIST1* expression (ref. Low) + 1.226×T stage (ref. T1-T2) + 1.778×M stage (ref. M0) + 2.515×AJCC stage (ref. I-II) + 1.954×ISUP grade (ref. 1-2) for OS. The Kaplan–Meier method was used to determine the significant survival outcomes (PFS: *p*<0.0001; OS: *p*<0.0001), shown in Figure 4A, 4B. Meanwhile, ROC curves were generated to validate the ability of the logistic model to predict prognosis. The AUC index for the integrated model were 0.886 (*p*<0.001) for PFS (Figure 4C) and 0.814 for OS (*p*<0.001) (Figure 4D). To further verify the practical value of the model, ROC curves were constructed to perform external validation using clinicopathological parameters and mRNA expression profiles from TCGA cohort. The AUC index for the integrated model were 0.720 (*p*<0.001) for PFS (Figure 4E) and 0.684 for OS (*p*<0.001) (Figure 4F).

**Table 2:** Univariate and multivariate Cox regression model in predicting progression-free and overall survival of clear cell renal cell carcinoma.

**Characteristic**	**Progression-free survival of FUSCC cohort**				**Overall survival of FUSCC cohort**			
	**Univariate model**		**Multivariate model**		**Univariate model**		**Multivariate model**	
	**HR (95% CI)**	***p*-value**	**HR (95% CI)**	***p*-value**	**HR (95% CI)**	***p*-value**	**HR (95% CI)**	***p*-value**
**Age**	1.163 (0.874-1.548)	0.300			1.334 (0.949-1.875)	0.097		
**Laterality**								
Left	Reference				Reference			
Right	1.053 (0.795-1.393)	0.720			1.030 (0.735-1.443)	0.886		
**ISUP grade**							
I-II	Reference		Reference		Reference		Reference	
III-IV	3.019 (2.225-4.098)	<0.001	2.013 (1.454-2.786)	<0.001	3.466 (2.364-5.083	<0.001	1.954 (1.286-2.970)	0.002
**Gender**								
Male	Reference				Reference			
Female	0.927 (0.685-1.255)	0.625			1.047 (0.732-1.498)	0.801		
**AJCC Stage**	4.285 (3.205-5.729)	<0.001	2.021 (1.357-3.010)	0.001	6.289 (4.440-8.907)	<0.001	2.515 (1.575-4.016)	<0.001
**pT stage**								
T1-T2	Reference		Reference		Reference		Reference	
T3-T4	4.285 (3.205-5.729)	<0.001	1.274 (1.087-1.492)	0.003	1.297 (1.183-1.423)	<0.001	1.226 (1.017-1.477)	0.032
**pN stage**								
N0	Reference		Reference		Reference		Reference	
N1	3.800 (2.670-5.408)	<0.001	1.372 (0.879-2.142)	0.164	4.869 (3.314-7.154)	<0.001	1.396 (0.860-2.266)	0.177
**M stage**								
M0	Reference		Reference		Reference		Reference	
M1	6.308 (4.354-9.138)	<0.001	1.919 (1.190-3.094)	0.008	7.664 (5.160-11.382)	<0.001	1.778 (1.065-2.970)	0.028
**CDH1 expression**							
Low	Reference		Reference		Reference		Reference	
High	0.708 (0.532-0.941)	0.017	0.708 (0.513-0.977)	0.035	0.466 (0.324-0.670)	<0.001	0.564 (0.379-0.837)	0.005
**CDH2 expression**							
Low	Reference				Reference			
High	0.918 (0.694-1.215)	0.550			0.753 (0.536-1.060)	0.104		
**SNAI1 expression**							
Low	Reference		Reference		Reference		Reference	
High	1.691 (1.277-2.240)	<0.001	1.360 (1.013-1.828)	0.041	2.183 (1.556-3.062)	<0.001	1.532 (1.062-2.211)	0.022
**SNAI2 expression**							
Low	Reference		Reference		Reference		Reference	
High	1.495 (1.125-1.988)	0.006	1.207 (0.898-1.621)	0.212	1.875 (1.317-2.668)	<0.001	1.227 (0.849-1.773)	0.277
**VIM expression**							
Low	Reference		Reference		Reference		Reference	
High	4.406 (3.237-5.998)	<0.001	1.905 (1.275-2.845)	0.002	4.940 (3.332-7.325)	<0.001	1.804 (1.042-3.122)	0.035
**TWIST1 expression**							
Low	Reference		Reference		Reference		Reference	
High	3.121 (2.310-4.218)	<0.001	2.179 (1.494-3.180)	<0.001	2.828 (1.963-4.075)	<0.001	1.714 (1.088-2.700)	0.020

Abbreviation: FUSCC: Fudan University Shanghai Cancer Center; ISUP: The International Society of Urological Pathology; AJCC: American Joint Committee on Cancer.

### EMT markers and ccRCC microenvironment

**Figure 5:**
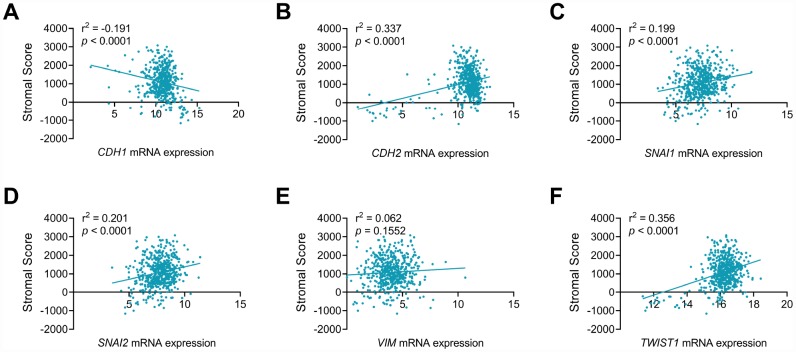
**CDH1, CDH2, SNAI1, SNAI2 and TWIST1 significantly involved in stromal process of ccRCC tumor environment.** EMT makers, including CDH1 (**A**), CDH2 (**B**), SNAI1 (**C**), SNAI2 (**D**), TWIST1 (**F**), but not VIM (**E**), showed significant correlation with stromal process in ccRCC environments (*p*<0.001). In addition, CDH1 showed negative association with stromal score (r^2^=-0.191), while stromal score positively correlated CDH2 (r^2^=0.337), SNAI1 (r^2^=0.199), SNAI2 (r^2^=0.201) and TWIST1 (r^2^=0.305) mRNA expression in ccRCC patients from TCGA cohort.

As seen in Figure 5, EMT makers, including CDH1 (Figure 5A), CDH2 (Figure 5B), SNAI1 (Figure 5C), SNAI2 (Figure 5D) and TWIST1 (Figure 5F), showed significant correlation with stromal process in ccRCC environments (*p*<0.001). In addition, CDH1 showed negative association with stromal score (r^2^=-0.191), while stromal score positively correlated CDH2 (r^2^=0.337), SNAI1 (r^2^=0.199), SNAI2 (r^2^=0.201) and TWIST1 (r^2^=0.305) mRNA expression in ccRCC patients from TCGA cohort.

### Module analysis and functional annotation

**Figure 6:**
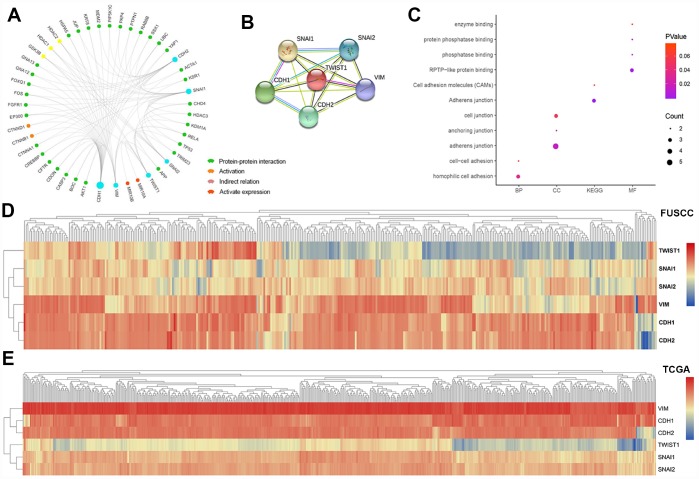
**Module analysis and functional annotations of the six EMT related gene *in* silico.** Protein-protein interaction (PPI), activation and indirect relation were predicted and displayed in association with sig EMT related genes (**A**). PPI network derived from active interaction sources was detailed illustrated with required interaction score equal 0.400 (**B**). GO and KEGG functional annotations analysis of *CDH1*, *CDH2*, *SNAI1*, *SNAI2*, *VIM*, *TWIST1* was enriched in hemophilic cell adhesion and cell-cell adhesion of biologic process, adherens junction, anchoring junction and cell junction of cellular component, RPTP-like protein binding, phosphatase binding, protein phosphatase binding and enzyme binding of molecular function. Participating upstream or downstream signaling pathways enrichment include adherens junction, cell adhesion molecules (CAMs) of KEGG pathways (**C**). Hierarchical partitioning using transcriptional expression profiles of *CDH1*, *CDH2*, *SNAI1*, *SNAI2*, *VIM*, *TWIST1* from FUSCC cohort (**D**). Hierarchical partitioning using transcriptional expression profiles of six hub genes from TCGA cohort was performed in the heat map (**E**).

In this study, PPI network, activation and indirect relation was predicted in Figure 6A and PPI network derived from active interaction sources was detailed illustrated with required interaction score equal 0.400 in Figure 6B. Function annotations of *CDH1, CDH2, SNAI1, SNAI2, VIM, TWIST1* was enriched in hemophilic cell adhesion and cell-cell adhesion of GO: BP, adherens junction, anchoring junction and cell junction of GO: CC, RPTP-like protein binding, phosphatase binding, protein phosphatase binding and enzyme binding of GO: MF. Participating upstream or downstream signaling pathways enrichment include adherens junction, cell adhesion molecules (CAMs) of KEGG pathways. The details were illustrated in Figure 6C. Hierarchical partitioning using transcriptional expression profiles of *CDH1, CDH2, SNAI1, SNAI2, VIM, TWIST1* from FUSCC cohort was performed in Figure 6D. Hierarchical partitioning using transcriptional expression profiles of six hub genes from TCGA cohort was performed in a heat map in Figure 6E.

### Significant genes and pathways obtained by GSEA

A total of 100 significant genes were obtained by GSEA with positive and negative correlation. Importantly, GSEA was used to perform hallmark analysis for *CDH1, CDH2, SNAI1, SNAI2, VIM, TWIST1*. Results suggested that the most involved significant pathways included apical junction, epithelial mesenchymal transition, estrogen response, hypoxia, kras signaling pathway up, inflammatory response, myogenesis, TNF-alpha signaling via NF-κB, etc. The details are shown in Supplementary Figure 2A–2F.

## DISCUSSION

The primary value of the expression of EMT related markers would be prediction of post-surgery prognosis when ccRCC patients seek guidance from clinicians. Although better understanding of the mechanism of ccRCC progression has been established with emerging evidence and studies, tumor stage and grade remain the most significant predictors of recurrence and survival. In other hand, clinicopathological characteristics also showed limitations after taking into consideration of the molecular diversity in ccRCC. In the study, we examined the differential expression of six key EMT markers in ccRCC tumors from Oncomine and TCGA databases. After further confirming their prognostic value of progression-free survival and overall survival of ccRCC patients from TCGA database, we validated it in ccRCC patients from FUSCC cohort. ROC curves were also generated to validate the ability of the logistic model to predict prognosis. Our results suggested that EMT related markers might be a good predictor of prognosis of ccRCC patients.

Since 2001, many prognosis models and nomograms for ccRCC have been developed and reported. Frank et al. proposed the SSIGN score in 2002, which predicted the outcome of unilateral ccRCC patients treated with radical nephrectomy and was composed of 1997 TNM stage, tumor size, nuclear grade, and histological tumor necrosis [8]. One year later in 2003, Leibovich et al. reported a risk stratification system, which aimed at progression to metastases after radical nephrectomy for clinically localized ccRCC patients [9]. The system included features of tumor stage, regional lymph node status, tumor size, nuclear grade, and histologic tumor necrosis. In 2004, Kim et al. first introduced molecular markers into prognostic model of ccRCC [10], which based on a combination of clinical and molecular predictors included metastasis status, T stage, Eastern Cooperative Oncology Group performance status, as well as immunohistochemically staining of p53, CA9, and vimentin. There was also another prognostic model, GRANT score, reported by Buti et al. recently [17]. In 2015, Rini et al. reported A 16-gene assay to predict recurrence after surgery in localized ccRCC patients [18], which did provide a more accurate and individualized risk assessment than previous clinicopathologic characteristics. However, it is currently beyond clinical access in terms of cost and complexity.

In the study, we focused on EMT related markers. *CDH1* (encoding E-cadherin) is one the main epithelial marker, whose downregulation will reinforce the destabilization of adherens junctions and is a hallmark of EMT [19]. *SNAI1* (encoding Snail) and *SNAI2* (encoding Slug) are two master regulatory transcription factors which can directly repress E-cadherin expression by binding to the specific E-boxes of E-cadherin's proximal promoter [20]. N-cadherin, however, contradictory to E-cadherin, is a marker of ongoing EMT [21]. Furthermore, cadherin switching (high N- cadherin and low E-cadherin) is essential for EMT, especially for cell behavior [22]. *TWIST1* (encoding TWIST1) is another important transcriptional activator that upregulates N-cadherin and downregulates E-cadherin [23]. *VIM* (encoding Vimentin) functions as a positive regulator of EMT, whose upregulation is thought to be a prerequisite for EMT induction [24]. Because of their crucial role in EMT process, we chose them as candidates in the study. Kaplan-Meier plots indicated that mRNA expressions of *CDH1*, *SNAI1*, *TWIST1*, and *VIM* were significant predictors for either progression-free survival or overall survival in both TCGA and FUSCC cohorts. After multivariate Cox regression analysis, these four EMT markers were confirmed to be independent prognostic factors for ccRCC patients in FUSCC cohort.

There are several studies which examined the prognostic value of these genes in RCC patients respectively, and the result was consistent with our finding. Decreased E-cadherin expression was associated with increased incidence of metastasis in RCC [25], as well as worse progression-free survival and overall survival of ccRCC patients [26]. Higher Snail expression was reported to be significantly associated with worse disease-free and disease-specific survival of the patients with RCC [14]. As for TWIST1, Rasti et al. reported that higher cytoplasmic expression of TWIST1 is associated with higher tumor grade and worse progression-free survival in ccRCC patients [27]. Vimentin expression was also reported to be an independent prognosticator of survival for localized RCC patients [28].

Due to emerging evidence showing that EMT as a clinically relevant mechanism of both tumor induction and progression, efforts have been made to develop new pharmacological therapies to target this process [29]. Unlike breast cancer [30, 31], colon cancer [32], lung cancer [33], and prostate cancer [34], EMT pathway inhibitors have not been applied in ccRCC. Combination of traditional target therapy and EMT related inhibitor may be a potential option for RCC patients. Besides, since RCC patients respond to immunotherapy, immune checkpoint inhibitor [35, 36] also have been approved for advanced RCC patients. Nonetheless, the median progression-free survival of advanced RCC patients was less than one year. Combining EMT related inhibitor with immune checkpoint inhibitor may also be promising to further improve patient prognosis.

There are several limitations of the study should be considered. First, this is a retrospective study of FUSCC cohort. Based on the current promising result, we aim to generate a more precise survival prediction panel of ccRCC in prospective cohorts using both tissue arrays and RNA-Seq. Second, the current study lacks of functional *in vivo* or *in vitro* experiments. In our further study, we plan to use our PDX (Patient Derived Xenograft) mouse model to test whether inhibition of EMT pathway would influence ccRCC progression. Third, there are surely other EMT markers which play crucial roles in ccRCC progression, such as β-catenin [37], MMP2 (matrix metalloproteinases) [14], MMP9 [14], and AXL [38]. At the moment, we haven’t screened all the potential EMT markers. We chose six key EMT markers and generated a preliminary prognostic model of ccRCC. We are planning to conduct a further screening of EMT related genes and generate a more detailed prognostic model. Besides, Since the racial difference between the TCGA (most were Caucasian, African-American) and FUSCC (Asian) cohorts existed, the prognostic model needs further validation across different populations.

## MATERIALS AND METHODS

### Oncomine database

In this study, transcriptional expression profiles of *CDH1, CDH2, SNAI1, SNAI2, VIM, TWIST1* in 20 different common neoplasms were obtained from Oncomine database using Oncomine online database (http://www.oncomine.com) [39]. Difference of transcriptional expression was compared by Student’s *t*-test. Cut-off of *p* value and fold change were as following: *p* value=0.01, Fold Change=1.5, gene rank=10%, Data type: mRNA.

### Patients from TCGA cohort and transcriptional expression profile

A total of 533 ccRCC patients with available RNA-sequence data from TCGA database were consecutively recruited in analyses [40]. The gene expression profile was measured experimentally using the Illumina HiSeq 2000 RNA Sequencing platform by the University of North Carolina TCGA genome characterization center. Level 3 data was downloaded from the Cancer Genomics Browser of the University of California Santa Cruz (https://genome-cancer.ucsc.edu/proj/site/hg Heatmap/). Differential transcriptional expression profiles of *CDH1, CDH2, SNAI1, SNAI2, VIM, TWIST1* was measured between ccRCC tumor samples and adjacent normal tissues, respectively.

### Statistical analysis of TCGA cohort

In this study, significant co-regulated network of genomic profiles of *CDH1, CDH2, SNAI1, SNAI2, VIM, TWIST1* and their frequently altered neighbor genes was constructed using cBioPortal, an open-access online resource accessing the TCGA genomics data [41]. Hierarchical partitioning was performed using transcriptional expression profiles of six hub genes in a heat map. Color gradients suggest high (red) or low (blue) expression level.

Among the 533 ccRCC patients, 516 patients had complete clinicopathologic information. Phenotype and expression profiles of hub genes in the 516 ccRCC patients from TCGA were analyzed and displayed. Survival comparison between distinct mRNA expressions groups of *CDH1, CDH2, SNAI1, SNAI2, VIM, TWIST1* were analyzed in ccRCC patients. The primary end point for patients was progression-free survival (PFS), and overall survival (OS) was the secondary end point, which was evaluated from the date of first therapy to the date of death or last follow-up. X-tile software was utilized to take the cut-off value of mRNA expression of six hub genes, in concordance of which overall participants were divided to two groups, respectively [42]. The follow-up duration was estimated using the Kaplan-Meier method with 95% confidence intervals (95% CI) and log-rank test in separate curves. Univariate and multivariate analysis were performed with Cox logistic regression models to find independent variables, including age at diagnosis, age at surgery, T stage (ref. T1-T2), N stage (ref. N0), M stage (ref. M0), AJCC (American Joint Committee on Cancer) stage (ref. I-II), ISUP (The International Society of Urological Pathology) grade (ref. 1-2), *CDH1* expression (ref. Low), *CDH2* expression (ref. Low), *SNAI1* expression (ref. Low), *SNAI2* expression (ref. Low), *VIM* expression (ref. Low) and *TWIST1* expression (ref. Low). All hypothetical tests were two-sided and *p*-values less than 0.05 were considered significant in all tests.

### Patients and variables from FUSCC cohort

To further validate prognostic implications of six hub genes in real world, a total of 367 ccRCC patients, who have undergone radical nephrectomy in the Department of Urology of Fudan University Shanghai Cancer Center (FUSCC) (Shanghai, China) from Aug. 2008 to Sept. 2016, were consecutively recruited in analyses, with electronic medical records or pathology reports available. Clinical and pathological parameters, specifically age at surgery, clinical manifestation, tumor laterality, TNM stage, ISUP grade classification were summarized. Tissue samples, including ccRCC and normal tissues, were collected during surgery and available from FUSCC tissue bank. A central review of pathology was performed by an experienced pathologist. Clinicopathological characteristics were obtained from electronic records. Patients were regularly followed up by telephone, mail, or in the clinic once every 3 months. All the study designs and test procedures were performed in accordance with the Helsinki Declaration II. The Ethics approval and consent to participate of the current study was approved and consented by the ethics committee of FUSCC.

### Real-time quantitative PCR analysis

Transcription level of six hub genes was measured using RT-PCR analysis in 367 ccRCC patients from FUSCC cohort. Total RNA sequence was extracted using TRIzol reagent (Invitrogen, Carlsbad, CA) from 367 paired tumor samples. Total RNA reverse-transcribed reaction was performed using the SuperScript First-Strand cDNA Synthesis System (Invitrogen, Carlsbad, CA). ABI Prism 7900 Sequence Detector (Applied Biosystems) was utilized to realize Real-time PCR reactions. Forward and reverse PCR primers of *CDH1, CDH2, SNAI1, SNAI2, VIM, TWIST1* were listed in Supplementary Table 1. According to SYBR Green PCR master mix (Applied Biosystems) manufacturer protocols, a total of 10μL reaction mixture was prepared for each test.

### Statistical analysis of FUSCC cohort

Kaplan-Meier method with 95% confidence intervals (95%CI) and log-rank test was applied to analyze different survival outcomes in separate curves in 367 ccRCC patients from FUSCC cohort. Univariate analyses were performed with Cox logistic regression models to find independent variables, including pT stage, pN stage, M stage, AJCC stage, and ISUP grade.

Integrated score was identified as sum of the weight of each significant hub gene. X-tile software was utilized to take the cut-off value. All hypothetical tests were two-sided and P-values less than 0.05 were considered significant in all tests. The receiver operating characteristic curve (ROC) was constructed by predicting the probability of a diagnosis being of high or low integrated score of significant hub gene expression. Area under curve (AUC) analysis was performed to determine the diagnostic ability. In addition, validation of diagnosis and prognostic value of integrated score was constructed using mRNA data from TCGA.

### EMT markers and ccRCC microenvironment

ESTIMATE algorithm, available from "estimate" R package, was obtained and utilized to measure to measure stromal components in ccRCC tumor microenvironment. Transcriptional expression levels of EMT markers and stromal scores were matched to sample identification names in the TCGA database. Pearson correlation coefficient analysis was used to reflect the degree of linear correlation between two random variables. The value of r is between -1 and 1. When the value is 1 or -1, the two random variables are completely negatively or positively correlated.

### Module analysis and functional annotations

In the present study, protein-protein interaction (PPI), activation, indirect relation and activation expression modular was predicted using Search Tool for the Retrieval of Interacting Genes (STRING; http://string-db.org) (version 10.0) online database was used to predict PPI network of DEGs and analyzing the functional interactions between proteins [43]. An interaction with a combined score >0.4 was considered statistically significant. Significant co-regulated network of six hub genes was constructed using cBioPortal. Subsequently, the gene ontology (GO): BP (biological process), GO: CC (cellular component), GO: MF (molecular function) and KEGG pathways analyses for a total of 48 genes in this module were performed using DAVID [44], and then visualized in bubble chart. Hierarchical partitioning using transcriptional expression profiles of *CDH1, CDH2, SNAI1, SNAI2, VIM, TWIST1* from FUSCC cohort was performed in a heat map. Color gradients suggest high (red) or low (blue) expression level.

### Data processing of gene set enrichment analysis (GSEA)

TCGA database were implemented with GSEA method using the Category version 2.10.1 package. For each separate analysis, Student’s-t-test statistical score was performed in consistent pathways and the mean of the differential expression genes was calculated. A permutation test with 1000 times was used to identify the significantly changed pathways. The adjusted P values (adj. P) using Benjamini and Hochberg (BH) false discovery rate (FDR) method by default were applied to correct the occurrence of false positive results [45]. The significant related genes were defined with an adj. P less than 0.01 and FDR less than 0.25. Statistical analysis and graphical plotting were conducted using R software (Version 3.3.2).

### Summary

We preliminarily tested six key EMT markers and validated their prognostic value in both TCGA cohort and USCC cohort. Among these six EMT markers, *CDH1*, *SNAI1*, *VIM*, and *TWIST1* were found to be independent PFS and OS of ccRCC patients.

## Supplementary Material

Supplementary Figures

Supplementary Table 1

## References

[1] Siegel RL, Miller KD, Jemal A. Cancer statistics, 2019. CA Cancer J Clin. 2019; 69:7–34. 10.3322/caac.21551 30620402

[2] Chen W, Zheng R, Baade PD, Zhang S, Zeng H, Bray F, Jemal A, Yu XQ, He J. Cancer statistics in China, 2015. CA Cancer J Clin. 2016; 66:115–32. 10.3322/caac.21338 26808342

[3] Baldewijns MM, van Vlodrop IJ, Schouten LJ, Soetekouw PM, de Bruïne AP, van Engeland M. Genetics and epigenetics of renal cell cancer. Biochim Biophys Acta. 2008; 1785:133–55. 10.1016/j.bbcan.2007.12.002 18187049

[4] Kane CJ, Mallin K, Ritchey J, Cooperberg MR, Carroll PR. Renal cell cancer stage migration: analysis of the National Cancer Data Base. Cancer. 2008; 113:78–83. 10.1002/cncr.23518 18491376

[5] Choueiri TK, Motzer RJ. Systemic Therapy for Metastatic Renal-Cell Carcinoma. N Engl J Med. 2017; 376:354–66. 10.1056/NEJMra1601333 28121507

[6] Patel HD, Kates M, Pierorazio PM, Hyams ES, Gorin MA, Ball MW, Bhayani SB, Hui X, Thompson CB, Allaf ME. Survival after diagnosis of localized T1a kidney cancer: current population-based practice of surgery and nonsurgical management. Urology. 2014; 83:126–32. 10.1016/j.urology.2013.08.088 24246317 PMC3892770

[7] Frank I, Blute ML, Cheville JC, Lohse CM, Weaver AL, Leibovich BC, Zincke H. A multifactorial postoperative surveillance model for patients with surgically treated clear cell renal cell carcinoma. J Urol. 2003; 170:2225–32. 10.1097/01.ju.0000095541.10333.a7 14634384

[8] Frank I, Blute ML, Cheville JC, Lohse CM, Weaver AL, Zincke H. An outcome prediction model for patients with clear cell renal cell carcinoma treated with radical nephrectomy based on tumor stage, size, grade and necrosis: the SSIGN score. J Urol. 2002; 168:2395–400. 10.1016/S0022-5347(05)64153-5 12441925

[9] Leibovich BC, Blute ML, Cheville JC, Lohse CM, Frank I, Kwon ED, Weaver AL, Parker AS, Zincke H. Prediction of progression after radical nephrectomy for patients with clear cell renal cell carcinoma: a stratification tool for prospective clinical trials. Cancer. 2003; 97:1663–71. 10.1002/cncr.11234 12655523

[10] Kim HL, Seligson D, Liu X, Janzen N, Bui MH, Yu H, Shi T, Figlin RA, Horvath S, Belldegrun AS. Using protein expressions to predict survival in clear cell renal carcinoma. Clin Cancer Res. 2004; 10:5464–71. 10.1097/01.ju.0000154351.37249.f0 15328185

[11] Nieto MA, Huang RY, Jackson RA, Thiery JP. Emt: 2016. Cell. 2016; 166:21–45. 10.1016/j.cell.2016.06.028 27368099

[12] Thiery JP, Sleeman JP. Complex networks orchestrate epithelial-mesenchymal transitions. Nat Rev Mol Cell Biol. 2006; 7:131–42. 10.1038/nrm1835 16493418

[13] Thiery JP, Lim CT. Tumor dissemination: an EMT affair. Cancer Cell. 2013; 23:272–73. 10.1016/j.ccr.2013.03.004 23518345

[14] Mikami S, Katsube K, Oya M, Ishida M, Kosaka T, Mizuno R, Mukai M, Okada Y. Expression of Snail and Slug in renal cell carcinoma: e-cadherin repressor Snail is associated with cancer invasion and prognosis. Lab Invest. 2011; 91:1443–58. 10.1038/labinvest.2011.111 21808237

[15] Harada K, Miyake H, Kusuda Y, Fujisawa M. Expression of epithelial-mesenchymal transition markers in renal cell carcinoma: impact on prognostic outcomes in patients undergoing radical nephrectomy. BJU Int. 2012; 110:E1131–37. 10.1111/j.1464-410X.2012.11297.x 22712620

[16] Kallakury BV, Karikehalli S, Haholu A, Sheehan CE, Azumi N, Ross JS. Increased expression of matrix metalloproteinases 2 and 9 and tissue inhibitors of metalloproteinases 1 and 2 correlate with poor prognostic variables in renal cell carcinoma. Clin Cancer Res. 2001; 7:3113–9. 11595703

[17] Buti S, Puligandla M, Bersanelli M, DiPaola RS, Manola J, Taguchi S, Haas NB. Validation of a new prognostic model to easily predict outcome in renal cell carcinoma: the GRANT score applied to the ASSURE trial population. Ann Oncol. 2017; 28:2747–53. 10.1093/annonc/mdx492 28945839 PMC5815563

[18] Rini B, Goddard A, Knezevic D, Maddala T, Zhou M, Aydin H, Campbell S, Elson P, Koscielny S, Lopatin M, Svedman C, Martini JF, Williams JA, et al. A 16-gene assay to predict recurrence after surgery in localised renal cell carcinoma: development and validation studies. Lancet Oncol. 2015; 16:676–85. 10.1016/S1470-2045(15)70167-1 25979595

[19] Lamouille S, Xu J, Derynck R. Molecular mechanisms of epithelial-mesenchymal transition. Nat Rev Mol Cell Biol. 2014; 15:178–96. 10.1038/nrm3758 24556840 PMC4240281

[20] Seki K, Fujimori T, Savagner P, Hata A, Aikawa T, Ogata N, Nabeshima Y, Kaechoong L. Mouse Snail family transcription repressors regulate chondrocyte, extracellular matrix, type II collagen, and aggrecan. J Biol Chem. 2003; 278:41862–70. 10.1074/jbc.M308336200 12917416 PMC2253659

[21] Wheelock MJ, Shintani Y, Maeda M, Fukumoto Y, Johnson KR. Cadherin switching. J Cell Sci. 2008; 121:727–35. 10.1242/jcs.000455 18322269

[22] Maeda M, Johnson KR, Wheelock MJ. Cadherin switching: essential for behavioral but not morphological changes during an epithelium-to-mesenchyme transition. J Cell Sci. 2005; 118:873–87. 10.1242/jcs.01634 15713751

[23] Leptin M. twist and snail as positive and negative regulators during Drosophila mesoderm development. Genes Dev. 1991; 5:1568–76. 10.1101/gad.5.9.1568 1884999

[24] Ivaska J. Vimentin: central hub in EMT induction? Small GTPases. 2011; 2:51–53. 10.4161/sgtp.2.1.15114 21686283 PMC3116616

[25] Katagiri A, Watanabe R, Tomita Y. E-cadherin expression in renal cell cancer and its significance in metastasis and survival. Br J Cancer. 1995; 71:376–79. 10.1038/bjc.1995.76 7841055 PMC2033607

[26] Harb OA, Elfeky MA, El Shafaay BS, Taha HF, Osman G, Harera IS, Gertallah LM, Abdelmonem DM, Embaby A. SPOP, ZEB-1 and E-cadherin expression in clear cell renal cell carcinoma (cc-RCC): Clinicopathological and prognostic significance. Pathophysiology. 2018; 25:335–345. 10.1016/j.pathophys.2018.05.004 29801752

[27] Rasti A, Madjd Z, Abolhasani M, Mehrazma M, Janani L, Saeednejad Zanjani L, Asgari M. Cytoplasmic expression of Twist1, an EMT-related transcription factor, is associated with higher grades renal cell carcinomas and worse progression-free survival in clear cell renal cell carcinoma. Clin Exp Med. 2018; 18:177–90. 10.1007/s10238-017-0481-2 29204790

[28] Sabo E, Miselevich I, Bejar J, Segenreich M, Wald M, Moskovitz B, Nativ O. The role of vimentin expression in predicting the long-term outcome of patients with localized renal cell carcinoma. Br J Urol. 1997; 80:864–68. 10.1046/j.1464-410X.1997.00489.x 9439398

[29] Rask-Andersen M, Almén MS, Schiöth HB. Trends in the exploitation of novel drug targets. Nat Rev Drug Discov. 2011; 10:579–90. 10.1038/nrd3478 21804595

[30] Gjerdrum C, Tiron C, Høiby T, Stefansson I, Haugen H, Sandal T, Collett K, Li S, McCormack E, Gjertsen BT, Micklem DR, Akslen LA, Glackin C, Lorens JB. Axl is an essential epithelial-to-mesenchymal transition-induced regulator of breast cancer metastasis and patient survival. Proc Natl Acad Sci USA. 2010; 107:1124–29. 10.1073/pnas.0909333107 20080645 PMC2824310

[31] Holland SJ, Pan A, Franci C, Hu Y, Chang B, Li W, Duan M, Torneros A, Yu J, Heckrodt TJ, Zhang J, Ding P, Apatira A, et al. R428, a selective small molecule inhibitor of Axl kinase, blocks tumor spread and prolongs survival in models of metastatic breast cancer. Cancer Res. 2010; 70:1544–54. 10.1158/0008-5472.CAN-09-2997 20145120

[32] Lai W, Liu L, Zeng Y, Wu H, Xu H, Chen S, Chu Z. KCNN4 channels participate in the EMT induced by PRL-3 in colorectal cancer. Med Oncol. 2013; 30:566. 10.1007/s12032-013-0566-z 23572150

[33] Xie M, Zhang L, He CS, Xu F, Liu JL, Hu ZH, Zhao LP, Tian Y. Activation of Notch-1 enhances epithelial-mesenchymal transition in gefitinib-acquired resistant lung cancer cells. J Cell Biochem. 2012; 113:1501–13. 10.1002/jcb.24019 22173954

[34] Nanta R, Kumar D, Meeker D, Rodova M, Van Veldhuizen PJ, Shankar S, Srivastava RK. NVP-LDE-225 (Erismodegib) inhibits epithelial-mesenchymal transition and human prostate cancer stem cell growth in NOD/SCID IL2Rγ null mice by regulating Bmi-1 and microRNA-128. Oncogenesis. 2013; 2:e42. 10.1038/oncsis.2013.5 23567619 PMC3641359

[35] Motzer RJ, Escudier B, McDermott DF, George S, Hammers HJ, Srinivas S, Tykodi SS, Sosman JA, Procopio G, Plimack ER, Castellano D, Choueiri TK, Gurney H, et al, and CheckMate 025 Investigators. Nivolumab versus Everolimus in Advanced Renal-Cell Carcinoma. N Engl J Med. 2015; 373:1803–13. 10.1056/NEJMoa1510665 26406148 PMC5719487

[36] Motzer RJ, Tannir NM, McDermott DF, Arén Frontera O, Melichar B, Choueiri TK, Plimack ER, Barthélémy P, Porta C, George S, Powles T, Donskov F, Neiman V, et al, and CheckMate 214 Investigators. Nivolumab plus Ipilimumab versus Sunitinib in Advanced Renal-Cell Carcinoma. N Engl J Med. 2018; 378:1277–90. 10.1056/NEJMoa1712126 29562145 PMC5972549

[37] Krabbe LM, Westerman ME, Bagrodia A, Gayed BA, Darwish OM, Haddad AQ, Khalil D, Kapur P, Sagalowsky AI, Lotan Y, Margulis V. Dysregulation of β-catenin is an independent predictor of oncologic outcomes in patients with clear cell renal cell carcinoma. J Urol. 2014; 191:1671–77. 10.1016/j.juro.2013.11.052 24291548

[38] Stone L. Kidney cancer: AXL expression predicts prognosis. Nat Rev Urol. 2017; 14:700. 10.1038/nrurol.2017.186 29116257

[39] Rhodes DR, Yu J, Shanker K, Deshpande N, Varambally R, Ghosh D, Barrette T, Pandey A, Chinnaiyan AM. ONCOMINE: a cancer microarray database and integrated data-mining platform. Neoplasia. 2004; 6:1–6. 10.1016/S1476-5586(04)80047-2 15068665 PMC1635162

[40] Tomczak K, Czerwińska P, Wiznerowicz M. The Cancer Genome Atlas (TCGA): an immeasurable source of knowledge. Contemp Oncol (Pozn). 2015; 19:A68–77. 10.5114/wo.2014.47136 25691825 PMC4322527

[41] Gao J, Aksoy BA, Dogrusoz U, Dresdner G, Gross B, Sumer SO, Sun Y, Jacobsen A, Sinha R, Larsson E, Cerami E, Sander C, Schultz N. Integrative analysis of complex cancer genomics and clinical profiles using the cBioPortal. Sci Signal. 2013; 6:pl1. 10.1126/scisignal.2004088 23550210 PMC4160307

[42] Camp RL, Dolled-Filhart M, Rimm DL. X-tile: a new bio-informatics tool for biomarker assessment and outcome-based cut-point optimization. Clin Cancer Res. 2004; 10:7252–59. 10.1158/1078-0432.CCR-04-0713 15534099

[43] Franceschini A, Szklarczyk D, Frankild S, Kuhn M, Simonovic M, Roth A, Lin J, Minguez P, Bork P, von Mering C, Jensen LJ. STRING v9.1: protein-protein interaction networks, with increased coverage and integration. Nucleic Acids Res. 2013; 41:D808–15. 10.1093/nar/gks1094 23203871 PMC3531103

[44] Huang DW, Sherman BT, Tan Q, Collins JR, Alvord WG, Roayaei J, Stephens R, Baseler MW, Lane HC, Lempicki RA. The DAVID Gene Functional Classification Tool: a novel biological module-centric algorithm to functionally analyze large gene lists. Genome Biol. 2007; 8:R183. 10.1186/gb-2007-8-9-r183 17784955 PMC2375021

[45] Subramanian A, Tamayo P, Mootha VK, Mukherjee S, Ebert BL, Gillette MA, Paulovich A, Pomeroy SL, Golub TR, Lander ES, Mesirov JP. Gene set enrichment analysis: a knowledge-based approach for interpreting genome-wide expression profiles. Proc Natl Acad Sci USA. 2005; 102:15545–50. 10.1073/pnas.0506580102 16199517 PMC1239896

