# Supplementary material for: Prognostic value of epithelial-mesenchymal transition markers in clear cell renal cell carcinoma
Source: Aging (Albany NY). 2020 Jan 8;12(1):866–83. doi: 10.18632/aging.102660 (PMC6977664; doi:10.18632/aging.102660)
Supplement: Supplementary Figures [file aging-12-102660-s002..pdf]

SUPPLEMENTARY FIGURES

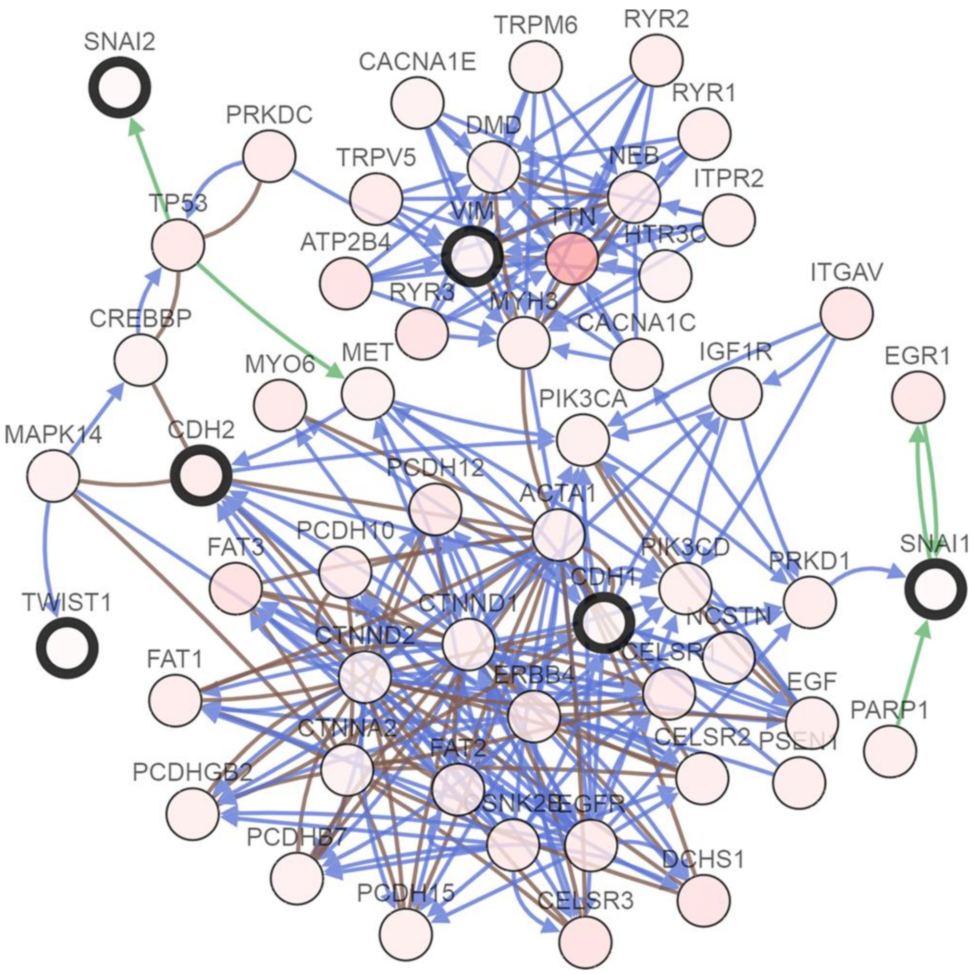

Supplementary Figure 1. Screening for neighbor genes and co-expression of six hub genes in transcription expression. Co-regulated network of *CDH1*, *CDH2*, *SNAI1*, *SNAI2*, *VIM*, *TWIST1* and their 49 frequently genetic altered neighbor genes was integrated and constructed using cBioPortal in transcriptional level.

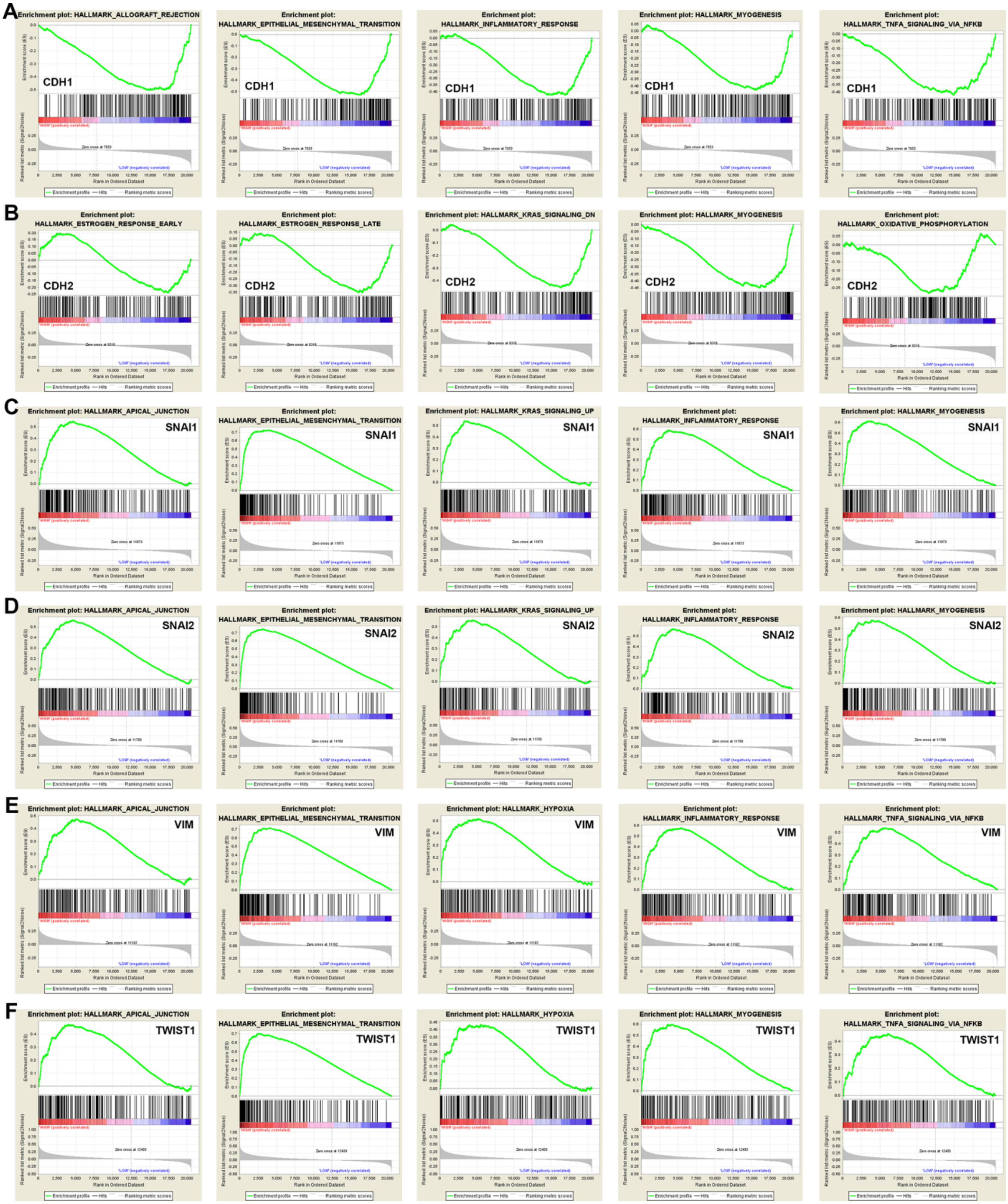

Supplementary Figure 2. GSEA was used to perform hallmark analysis for *CDH1*, *CDH2*, *SNAI1*, *SNAI2*, *VIM*, *TWIST1*. It suggested that the most involved significant pathways included apical junction, epithelial mesenchymal transition, estrogen response, hypoxia, kras signaling pathway up, inflammatory response, myogenesis, TNF-alpha signaling via NF-κB, etc.
