# Supplementary material for: Prognostic value of epithelial-mesenchymal transition markers in clear cell renal cell carcinoma
Source: Aging (Albany NY). 2020 Jan 8;12(1):866–83. doi: 10.18632/aging.102660 (PMC6977664; doi:10.18632/aging.102660)
Supplement: Supplementary Table 1 [file aging-12-102660-s001..pdf]

SUPPLEMENTARY TABLE

Supplementary Table 1. Information of primers used in the study.

Gene_ID	Sequence
CDH1	F: CGAGAGCTACACGTTCACGG<br>R: GGGTGTCGAGGGAAAAATAGG
CDH2	F: AGCCAACCTTAACTGAGGAGT<br>R: GGCAAGTTGATTGGAGGGATG
SNAI1	F: TCGGAAGCCTAACTACAGCGA<br>R: AGATGAGCATTGGCAGCGAG
SNAI2	F: CGAACTGGACACACATACAGTG<br>R: CTGAGGATCTCTGGTTGTGGT
VIM	F: GACGCCATCAACACCGAGTT<br>R: CTTTGTCGTTGGTTAGCTGGT
TWIST1	F: GTCCGCAGTCTTACGAGGAG<br>R: GCTTGAGGGTCTGAATCTTGCT
